# Impact of Differing Language Background Exposures on Bayley-III Language Assessment in a National Cohort of Children Born Less than 29 Weeks’ Gestation

**DOI:** 10.3390/children9071048

**Published:** 2022-07-14

**Authors:** Natalie Hoi-Man Chan, Anne Synnes, Ruth E. Grunau, Lindsay Colby, Julie Petrie, Tracy Elfring, Lindsay Richter, Leonora Hendson, Rudaina Banihani, Thuy Mai Luu, on behalf of the Canadian Neonatal Follow-Up Network Investigators

**Affiliations:** 1 Neonatal Follow-Up Program, British Columbia Women’s Hospital and Health Centre, Vancouver, BC V6H 3V4, Canada; anne.synnes@bcchr.ca (A.S.); rgrunau@bcchr.ca (R.E.G.); lcolby@cw.bc.ca (L.C.); jpetrie@cw.bc.ca (J.P.); tracy.elfring1@cw.bc.ca (T.E.); 2 Department of Paediatrics, University of British Columbia, Vancouver, BC V6H 3V4, Canada; lrichter@bcchr.ca; 3 Department of Pediatrics, University of California, San Francisco, CA 94143, USA; 4 Department of Pediatrics, Cumming School of Medicine, University of Calgary, Calgary, AB T2N 1N4, Canada; leonora.hendson@albertahealthservices.ca; 5 DAN Women and Babies Program, Sunnybrook Health Sciences Centre, Toronto, ON M4N 3M5, Canada; rudaina.banihani@sunnybrook.ca; 6 Department of Pediatrics, University of Toronto, Toronto, ON M5G 1X8, Canada; 7 Department of Pediatrics, Centre Hospitalier Universitaire Sainte-Justine, University of Montreal, Montreal, QC H3T 1C5, Canada

**Keywords:** prematurity, language, Bayley, neurodevelopment

## Abstract

Preterm infants are at risk for adverse neurodevelopmental outcomes, especially language delay. Preterm infants < 29 weeks’ gestational age, cared for in Canadian Neonatal Follow-Up Network affiliated hospitals, were assessed between 18 to 21 months corrected age using the Bayley-III. Bayley-III Language Composite Scores were compared using univariate and multivariate analyses for children in three primary language groups: English, French and other. 6146 children were included. The primary language at home was English, French or another language for 3708 children (60%), 1312 children (21%) and 1126 children (18%), respectively, and overall, 44% were exposed to two or more languages at home. Univariate analysis showed that primary language was associated with lower Bayley-III Language scores; however, multivariate analyses demonstrated that neither primary language nor language of administration were significantly associated with lower language scores when adjusted for gestational age, other developmental delays and sociodemographic factors, but multiple language exposure was. Sociodemographic and other factors are more important in determining language development than primary language at home. Further studies are needed to examine the association between exposure to multiple languages and lower Bayley-III language scores in preterm infants.

## 1. Introduction

For children born preterm, language delay is common [1,2,3], affecting about one third of children born at less than 29 weeks’ gestation in Canada [4]. Language and communication are integral to social development, academic success, emotional and mental well-being [5,6,7,8]. Early diagnosis and intervention have been shown to benefit children born preterm [9,10,11,12]. Studying language delay in populations of children born preterm is therefore important for clinical management, health service provision and quality improvement. Neonatal follow-up programs routinely assess development of at-risk children with the goal of early diagnosis of delays to initiate early and timely interventions [13].

The Bayley Scales of Infant and Toddler Development, 3rd edition (Bayley-III) [14] is one of the most frequently used tools to evaluate language in infants and toddlers [15], providing a Composite Language score that assesses both receptive and expressive language skills. The Bayley-III has been officially validated in English, but it has been used internationally in non-English speaking countries, typically using a bilingual examiner or an interpreter [16]. Previous studies have raised the potential concern for unreliable language component testing, specifically for infants whose primary language is not English [17,18]. To evaluate the effects of the primary language spoken at home and the language of administration, potential confounding influences such as sociodemographic factors and multilingualism need to be considered. Canada is a multicultural country with two official languages, English and French. The great diversity in language and culture across the country presents an opportunity to better understand the effect of language environments on assessment of language outcomes in the preterm population.

Our first research aim was to compare, in a Canadian cohort of children born at less than 29 weeks’ gestational age (GA) assessed at a targeted corrected age of 18–21 months, whether Bayley-III language outcomes differ between children with English, French and other primary languages at home, adjusted for other risk factors. Our second aim was to explore whether administering the Bayley-III in French or with an interpreter affected language scores compared to administering in English.

## 2. Methods

### 2.1. Participants

This is a retrospective cohort study of preterm infants born less than 29 weeks’ GA between 1 April 2009 and 30 September 2016 and cared for in neonatal intensive care units (NICUs) across all of Canada that are affiliated with the Canadian Neonatal Follow-Up Network (CNFUN). Children with Bayley-III Language Composite scores and an identified primary language at home were included in the study.

**Figure 1:**
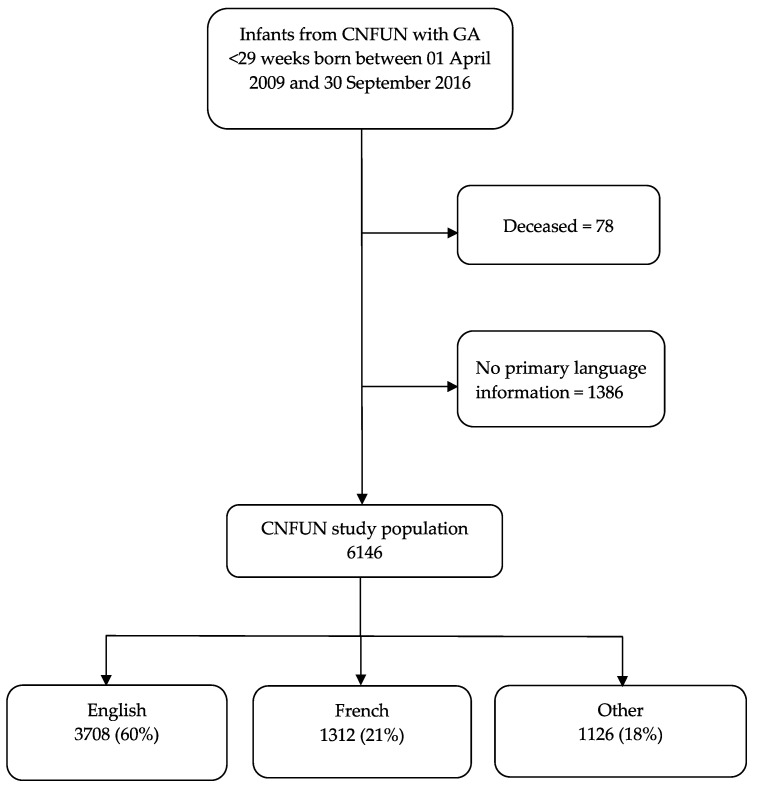
Participant flow diagram divided into groups of primary language at home.

A total of 7610 preterm infants (<29 weeks’ GA) were born during the study period and cared for at a CNFUN-affiliated NICU. The 78 deaths after NICU discharge and 1386 infants with no primary language information were excluded, leaving 6146 children for analysis in this study. Following STrengthening the Reporting of OBservational studies in Epidemiology (STROBE) guidelines [19], Figure 1 displays the flow of participants.

Research ethics board approval was obtained through the University of British Columbia and the Children’s and Women’s Health Center of British Columbia (H15-02774). Individual hospital research ethics boards approved the data collection. Consent from legal guardians was obtained where required by site research ethics boards.

### 2.2. Materials

CNFUN is a voluntary collaboration of neonatal follow-up programs across Canada that work together to improve the care of preterm infants through integrated data collection, research and knowledge translation (Table S1). CNFUN created a comprehensive standardized assessment of preterm infants <29 weeks’ GA at a targeted corrected age of 18 to 21 months. Data were uploaded to a central database with error checking. A manual of definitions and procedures was used [20]. Primary language at home, number of exposed languages and socioeconomic variables were elicited from parents as part of standardized history taking during visits. The neurodevelopmental assessment has been previously described [4].

The Bayley-III [14] is a standardized developmental assessment that is widely used to evaluate neurodevelopmental outcomes in children between 1 and 42 months. Cognitive, Language and Motor domains are assessed in person in one session of about 60 to 90 min in duration. Language items in toddlers less than 21 months are primarily vocabulary items with grammar and language structure evaluated more in older children. Two additional domains can be assessed by questionnaire, social-emotional and adaptive behaviour. In this study, only the cognitive, language and motor domains were assessed.

### 2.3. Procedures

The Cognitive, Language and Motor domains of the Bayley-III were administered by trained assessors during neonatal follow-up clinic visits at CNFUN sites. Children whose primary language at home was not English had either fluent bilingual examiners who administered their Bayley-III in French, or they had professional interpreters who translated the examiner’s instructions into their primary language. For families who declined an interpreter, the caregiver present for the assessment acted as the interpreter.

The primary outcome of interest was the Language Composite score on the Bayley-III. Bayley-III Composite scores, with a mean of 100 and a standard deviation of 15, were calculated for each assessed domain [14]. Low scores were defined as scores more than 1 standard deviation below the mean (i.e., composite score < 85). Hearing impairment was determined by history, chart review and/or physical exam. Deafness was defined as the prescription of hearing aids or cochlear implant. Experienced physicians completed the neurological examination with standard definitions to diagnose cerebral palsy (CP) [21].

### 2.4. Data Analyses

Descriptive statistics were used to summarize demographic and developmental characteristics of the study population. Participant characteristics were compared between three primary language groups: English, French and other. Frequency (percentage) and mean (standard deviation) or median (interquartile range) were reported for categorical and continuous variables, respectively. Differences among groups were assessed by Pearson Chi-square for categorical variables and ANOVA or Kruskal–Wallis test as appropriate for continuous variables.

Multivariable logistic regression analyses adjusted for potential confounders identified in the univariate analysis were conducted. The covariates adjusted included gestational age, Bayley-III Cognitive Composite score, Bayley-III Motor Composite score, rural residence (by postal code), use of hearing aids or cochlear implants, caregiver education and single caregiver status or employment status and exposure to multiple languages at home. Interaction effect was examined between covariates that may potentially be collinear. Data management and all statistical analyses were performed using SAS 9.4 (SAS Institute, Inc., Cary, NC, USA). A two-sided *p*-value of <0.05 was considered statistically significant.

## 3. Results

### 3.1. Sociodemographic and Language Group Characteristics

Data on sociodemographic characteristics, exposure to languages other than English and neurodevelopmental status are presented in Table 1. In this cohort, the primary language spoken at home was English for 3708 children (60%), French for 1312 children (21%) and another language for 1126 children (18%). Nearly half of the cohort (44%) were exposed to two or more languages at home, with children whose primary language was other being much more likely to be exposed to more than one language.

The mean gestational age was 26.3 weeks, which was similar across primary language groups. Primary caregiver education was significantly different between language groups, with non-completion of high school rates ranging from 7.9% in the English primary language group to 11.3% for other primary languages and 13.3% for the French group. Children whose primary language at home was English or French were much more likely to have at least one caregiver who was born in Canada. Self-reported caregiver ethnic group composition differed between primary language groups with a predominance of Asian ethnicity in families whose primary language was other and Caucasian ethnicity in families whose primary language was English or French. Childcare was more likely to be outside the home for children with French as a primary language.

### 3.2. Neurodevelopmental Outcomes by Language Groups

The proportion of all subjects with low Bayley-III Composite scores (standard scores < 85) was 36% for language, 21.5% for motor and 15.2% for cognitive. A significantly higher proportion of children with other primary language had lower language scores (43.7%) as well as cognitive scores (17.8%). Rates of definitive CP and proportion of children with low motor scores did not significantly differ between primary language groups. For the entire cohort, hearing impairment (sensorineural, conductive or mixed sensorineural/conductive and auditory neuropathy) was found in 4.6%, and 2.1% had a hearing aid or cochlear implant. In children with French as a primary language, conductive hearing loss was more common, but fewer had hearing aids or cochlear implant.

### 3.3. Bayley-III Administration Characteristics

When the Bayley-III was administered, a domain was omitted more frequently when the primary language was other (Table 2). For children whose primary language was other, the Bayley-III was administered in English 72.8% of the time, while a professional or parent interpreter translated 18.6% of the time (Table 3).

### 3.4. Univariate Analyses

In univariate analyses (Table 4), children had higher odds of a low Bayley-III Language Composite score (standard score < 85) if: their primary language was other, an interpreter was used, they were exposed to two or more languages at home, they were born at a lower gestational age, their caregivers had a lower level of education (a greater dose effect for primary caregiver), their caregiver was unemployed, or if they did not have at least one caregiver born in Canada. Children were also more likely to have lower language composite scores if they had a developmental delay with a low Bayley-III Cognitive score or Motor Composite score (<85).

### 3.5. Multivariate Analyses

In multivariate logistic regression (Table 5), the presence of another developmental delay or hearing impairment conferred the greatest odds for having a low Bayley-III Language Composite score. Low Bayley-III Language Composite score was significantly correlated with both caregiver education and single caregiver status but not with primary language at home. In the multivariate analysis assessing the language of administration (Table 6), there was no statistically significant difference if the Bayley-III was administered in English vs. French or with or without an interpreter. Being exposed to more than one language at home was associated with lower language scores when adjusted for sociodemographic factors and either primary language or language of administration. There was no interaction effect between the primary language and the number of languages exposed to at home. Additionally, there were no interaction effects between caregiver education or caregiver employment with the number of languages exposed to at home, nor with primary language at home.

## 4. Discussion

Our study identifies many factors associated with language delay in preterm infants born <29 weeks’ GA. Not surprisingly, having developmental delays in other domains such as cognitive and/or motor skills or significant hearing impairment were all highly associated with lower language scores [22,23,24,25]. Since language development is known to be strongly influenced by the social environment, the associations between lower language scores and caregiver characteristics such as lower education, unemployment and single caregiver status were also expected findings, which highlight the importance of ongoing equitable resources to support these higher-risk families [25].

### 4.1. Bayley-III Language Composite Scores and Primary Language at Home

The answer to our primary research question was that the primary language at home did not affect language scores when adjusted for sociodemographic variables in multivariate analyses, which suggests that sociodemographic characteristics rather than the primary language influence language development. This provides researchers and clinicians with the confidence that the Bayley-III is a useful measure of language development in non-English speaking toddlers when adjustment is made for sociodemographic factors.

Our study was not designed to evaluate the effects of linguistics on language development using the Bayley-III. However, consideration must be made for several important factors, including differences in morphology, syntax, semantics and pragmatics between languages and the normal processes of second language and dual language acquisition (e.g., impact of language dominance fluctuation, interference/transfer, etc.) [26]. Linguistics become increasingly important as children get older and their language matures. Economic, ethnic and cultural factors may affect the assessment of development [27,28], especially language. For instance, a child who does not recognize a commonly used word in English (e.g., foods, animals) may know other culturally appropriate words in their home language.

Although Bayley-III language scores may not fully represent a non-English speaking child’s abilities, they are a useful starting point to deepen our understanding of language development from both research and clinical perspectives. Additionally, they help identify children for more in-depth evaluation to see if they would benefit from early intervention. Children with lower Bayley-III scores can be further evaluated by a speech and language pathologist, who can examine additional factors that influence communication skills and help differentiate between a communication disorder and a language difference. These factors would include home language use and dialect, dual versus second language learning, non-verbal communication, etc. [26]. Additionally, monitoring executive function development and processing speed could help better understand whether they may be possible factors contributing to lower language scores.

### 4.2. Language of Administration of the Bayley-III

Administering the Bayley-III in French or with an interpreter compared to in English affected language scores on univariate analysis. However, in multivariate analyses, the language of administration did not significantly affect language scores. Although 18% of children in this cohort had a primary language other than English or French, the majority of these children with other primary language had the Bayley-III administered in English (72.8%), with only 18.6% administered with an interpreter. The relatively small number of children who were assessed with an interpreter may have influenced the ability of our study to detect a difference, but the adjusted odds ratios were low compared to the other variables we analyzed.

### 4.3. Multilingualism and Impact on Language Development

In this study, a secondary finding was that exposure to multiple languages is associated with lower language scores in preterm toddlers at corrected age of 18-21 months. A dose response effect was observed with exposure to more than two languages (adjusted odds ratio (aOR) 1.62 (95% confidence interval (CI): 1.18, 2.23) affecting Bayley-III Language Composite Scores more than exposure to two languages (aOR 1.45 (95% CI: 1.21, 1.74). Multilingualism is a risk factor for language delay in children born preterm [3,29], but there remains much uncertainty regarding how it contributes to poor language outcomes. Multilingual home environments were also previously found to be associated with lower cognitive scores in preterm infants [30].

Multilingualism may have a mixed impact on language development [31,32,33,34]. Our study highlights an important area for further investigation, as it is known that there are significant differences in the developing brain of term versus preterm infants. Young children who grow up in bilingual households usually are exposed to each language less and being bilingual may affect processing speed and efficiency [31]. However, there are some meta-language and cognitive benefits to being bilingual, such as ability to differentiate between languages and enhanced perceptual skills [31]. Unlike in term infants, where exposure to more languages is associated with gains in meta-language development [31,35,36,37], our results, similar to previous studies [17,30,38], indicate this may not be the case for preterm infants, which may point to vulnerability in the acquisition of language and communication skills as a result of preterm birth. There are likely differences in the way in which term versus preterm children develop language within a bilingual environment.

Where bilingual term children have shown gains in meta-language development, those born preterm may not be able to engage their metacognitive systems as well as their full-term bilingual counterparts to benefit from dual exposure at this point in their development. Altered brain development in very preterm infants and children is related to many functions involved in language development and may affect the capacity to benefit from exposure to a second or multiple languages. Thinner corpus callosum found in very preterm children is key to the exchange of interhemispheric information, which is central to speech and language processes [36]. Abnormal neonatal brain development, including white matter dysmaturation contributes to slower processing speed and poorer attention [37], which may also place a burden on second language acquisition [39]. Furthermore, white matter dysmaturation and altered functional connectivity in the preterm versus the term brain likely contribute to this potential differential impact of language environments on preterm compared to term-born infants [40]. As early as age 12 months, executive functions [41] such as cognitive flexibility and shifting are altered in preterm infants, which is another potential contributing factor to challenges of acquiring a second language.

Phonological awareness and perceptual narrowing are foundational skills in language development [40,42]. Jansson-Verkasalo et al. [42] examined the trajectory of phoneme differentiation between native and non-native languages in the early years. They demonstrated that preterm infants do not have decreased responses to non-native language phonemes after 12 months of life, unlike in term infants. Not being able to discriminate phonemes from different languages may make it more confusing for preterm infants to understand and differentiate words in different languages. This hindered early development of understanding of phonemes as building blocks of different words may contribute to delays in language skills and may be exacerbated when preterm infants are exposed to multiple languages in their environment.

Our results suggest that when working with preterm infants exposed to a multilingual environment, different strategies may be required to promote more optimal language development and the ability to learn different languages [43] more adeptly. This is particularly important in our increasingly multicultural multilingual world, where the home language may not be the dominant language in the social environment. More understanding of this phenomenon of impact of multiple language exposures will enable providers who care for children born preterm to be more specific in facilitating their language development while honouring their family’s background, cultural needs, and preferences.

### 4.4. Limitations

Given that CNFUN only recruits very preterm children, there was no full-term control group. Previous studies [3,44,45] have compared preterm and full-term control infant language abilities and have shown that preterm infants tend to have lower language skills. It remains unclear if there are other impacts of language environment on language development in preterm versus term infants.

In our study, it was not feasible to administer a “gold standard” validated culturally appropriate language assessment in the child’s primary language. The possibility that language abilities were underestimated in non-English/non-French speaking children cannot be excluded. Nonetheless, our study provides confidence that with adjustment for neurodevelopmental delays, gestational age and sociodemographic factors, the primary language at home is not a significant confounding variable.

Although we found an association between lower Bayley-III language scores for children exposed to a greater number of languages at home, it is important to be mindful that the scores themselves may not be truly reflective of the abilities of children with other primary languages. Our study is limited by the inherent challenge of adequately assessing language skills of children whose primary language is not the dominant language in which standardized tests like the Bayley-III are developed and validated. Using the Bayley-III, in fact, may over-identify children with language delays, and Bayley-III scores themselves may not provide enough information to assess what is really happening for each child.

## 5. Conclusions

This study found that several sociodemographic factors are associated with language exposure in young children. Adjustment for these factors eliminates primary language exposure as a determinant of Bayley-III language scores. Similarly, with adjustment for risk factors, we did not identify that using an interpreter and language of administration of the Bayley-III significantly affected Bayley-III Language Composite scores. These findings support the use of the Bayley-III in populations where the primary language is not English, which is reassuring for international researchers using the Bayley-III in other languages. We did find that exposure to multiple languages was associated with lower language scores; however, this result needs to be further studied.

## Figures and Tables

**Table 1:** Characteristics of preterm infants (born at <29 weeks’ GA) included in the study.

	Entire Cohort N = 6146	English N = 3708	French N = 1312	Other N = 1126	*p* Value
**Patient Variables**					
Gestational age, weeks, mean (SD)	26.3 (1.4)	26.3 (1.4)	26.4 (1.4)	26.3 (1.4)	0.13
**Socioeconomic Variables**					
Caregiver 1 level of education N (%):					
Did not complete high school	572 (9.7)	281 (7.9)	170 (13.3)	121 (11.3)	<0.01
Completed high school only	2007 (34.0)	1272 (35.8)	401 (31.3)	334 (31.2)	
Completed post-secondary	3320/5899 (56.3)	1996/3549 (56.2)	710/1281 (55.4)	614/1069 (57.4)	
Caregiver 2 level of education N (%):					
Did not complete high school	621 (11.5)	303 (9.4)	193 (16.2)	125 (12.2)	<0.01
Completed high school only	2089 (38.5)	1293 (40.2)	431 (36.3)	365 (35.8)	
Completed post-secondary	2714/5424 (50.0)	1618/3214 (50.3)	565/1189 (47.5)	531/1021 (52.0)	
Employed caregiver N (%)	5489/5997 (91.5)	3291/3604 (91.3)	1212/1300 (93.2)	986/1093 (90.2)	0.02
Single caregiver N (%)	407/6146 (6.6)	300/3708 (8.1)	69/1312 (5.3)	38/1126 (3.4)	<0.01
Number of people in the home, median (IQR)	4 (3, 5)	4 (3, 5)	4 (3, 4)	4 (3, 5)	<0.01
Number of adults in the home N (%):					
One	382 (7.5)	258 (8.7)	88 (7.6)	36 (3.7)	<0.01
Two	4085 (80.5)	2380 (80.4)	1002 (87.0)	703 (73.1)	
Three	319 (6.3)	191 (6.5)	40 (3.5)	88 (9.2)	
Four or more	288/5074 (5.7)	131/2960 (4.4)	22/1152 (1.9)	135/962 (14.0)	
Childcare N (%):					
In the home	793 (16.7)	480 (17.7)	115 (10.1)	198 (22.5)	<0.01
Outside the home	1638 (34.6)	780 (28.8)	697 (61.1)	161 (18.2)	
None	2303/4734 (48.7)	1451/2711 (53.5)	329/1141 (28.8)	523/882 (59.3)	
Rural residence	662/4934 (13.4)	379/2881 (13.2)	239/1113 (21.5)	44/940 (4.7)	<0.01
**Language Variables**					
Number of languages exposed to N (%):					
One	2742 (56.1)	1828 (65.1)	789 (70.1)	125 (13.1)	<0.01
Two	1827 (37.4)	850 (30.2)	274 (24.3)	703 (73.9)	
Three or more	318/4887 (6.5)	132/2810 (4.7)	63/1126 (5.6)	123/951 (12.9)	
At least one caregiver born in Canada N (%)	3835/5157 (74.4)	2576/2964 (86.9)	1069/1291 (82.8)	190/902 (21.1)	<0.01
Caregiver 1 ethnic group N (%):					
Caucasian	3238 (57.5)	2042 (61.4)	996 (77.0)	200 (19.8)	<0.01
Black	525 (9.3)	272 (8.2)	204 (15.8)	49 (4.9)	
Asian	796 (14.2)	323 (9.7)	21 (1.6)	452 (44.8)	
First Nations	200 (3.5)	166 (5.0)	10 (0.8)	24 (2.4)	
Other	296 (5.3)	91 (2.7)	51 (3.9)	154 (15.3)	
Unknown	571/5626 (10.2)	429/3323 (12.9)	12/1294 (0.9)	130/1009 (12.9)	
Caregiver 2 ethnic group N (%):					
Caucasian	3119 (59.6)	1970 (64.5)	945 (77.9)	204 (21.0)	<0.01
Black	457 (8.7)	234 (7.7)	177 (14.6)	46 (4.8)	
Asian	691 (13.2)	241 (7.9)	23 (1.9)	427 (44.1)	
First Nations	153 (2.9)	127 (4.2)	3 (0.3)	23 (2.4)	
Other	279 (5.3)	87 (2.8)	49 (4.0)	143 (14.8)	
Unknown	536/5235 (10.2)	394/3053 (12.9)	16/1213 (1.3)	126/969 (13.0)	
**Bayley-III Language**					
Language Composite Score, median (IQR)	91 (79, 100)	91 (79, 100)	91 (79, 100)	86 (74, 97)	<0.01
Language Composite < 85 N (%)	1960/5442 (36.0)	1151/3319 (34.7)	397/1181 (33.6)	412/942 (43.7)	<0.01
Language Composite < 70 N (%)	643/5442 (11.8)	383/3319 (11.5)	114/1181 (9.7)	146/942 (15.5)	<0.01
**Other Impairments**					
**Hearing N (%):**					
Sensorineural	75 (1.2)	51 (1.4)	9 (0.7)	15 (1.3)	<0.01
Conductive	156 (2.5)	67 (1.8)	71 (5.4)	18 (1.6)	
Mixed	10 (0.2)	6 (0.2)	3 (0.2)	1 (0.1)	
Auditory Neuropathy	29 (0.5)	16 (0.4)	2 (0.1)	11 (1.0)	
Unknown	40/6146 (0.7)	26/3708 (0.7)	10/1312 (0.8)	4/1126 (0.4)	
Hearing aids or cochlear implant	125/5957 (2.1)	89/3579 (2.5)	15/1288 (1.2)	21/1090 (1.9)	0.02
**Motor:**					
Definitive CP N (%)	387/6019 (6.4)	250/3619 (6.9)	73/1300 (5.6)	64/1100 (5.8)	0.17
Bayley-III Motor Composite, median (IQR)	94 (85, 100)	94 (85, 100)	94 (85, 100)	94 (85, 100)	0.05
Bayley-III Motor Composite < 85 N (%)	1164/5423 (21.5)	727/3309 (22.0)	231/1148 (20.1)	206/966 (21.3)	0.42
**Cognitive:**					
Bayley-III Cognitive, median (IQR)	95 (90, 105)	95 (90, 105)	95 (90, 105)	95 (85, 100)	<0.01
Bayley-III Cognitive < 85 N (%)	856/5638 (15.2)	528/3429 (15.4)	149/1202 (12.4)	179/1007 (17.8)	<0.01

Abbreviations: IQR, interquartile range; SD, standard deviation; CA, corrected age; CP, cerebral palsy.

**Table 2:** Frequency of administering the Bayley-III components by primary language groups.

	English or French N = 5020	Other N = 1126
Bayley-III Language administered, n (%)	4500 (89.6)	942 (83.7)
Bayley-III Cognitive administered, n (%)	4631 (92.3)	1007 (89.4)
Bayley-III Motor administered, n (%)	4457 (88.8)	966 (85.8)

**Table 3:** Primary language at home and language used for Bayley-III administration.

	Primary Language at Home		
Language of Bayley-III Administration	English N = 2705	French N = 995	Other N = 816
English, n (%)	2672 (98.8)	52 (5.2)	594 (72.8)
French, n (%)	24 (0.9)	932 (93.7)	70 (8.6)
Interpreter or parent interpreter, n (%)	9 (0.3)	11 (1.1)	152 (18.6)

**Table 4:** Factors affecting odds of Bayley-III Language Composite Scores < 85—univariate analysis.

Variable	Odds Ratio (95% CI ^1^)
English or French as a primary language	0.67 (0.58, 0.78)
Language of administration	
French vs. English	0.93 (0.80, 1.08)
Interpreter vs. English	1.41 (1.03, 1.93)
Number of languages exposed to (vs. one)	
Two	1.54 (1.35, 1.76)
More than two	1.69 (1.31, 2.18)
Gestational age	0.84 (0.80, 0.87)
Caregiver 1 education (vs. completed post-secondary)	
Did not complete high school	2.18 (1.80, 2.65)
Completed high school only	1.59 (1.41, 1.80)
Caregiver 2 education (vs. completed post-secondary)	
Did not complete high school	1.59 (1.31, 1.92)
Completed high school only	1.41 (1.24, 1.60)
One or two employed caregiver(s)	0.44 (0.36, 0.53)
Urban vs. rural residence	1.17 (0.97, 1.40)
At least one caregiver born in Canada	0.51 (0.44, 0.59)
Bayley-III Motor Composite Score < 85	0.93 (0.93, 0.94)
Bayley-III Cognitive Score < 85	0.90 (0.90, 0.91)

^1^ 95% confidence interval.

**Table 5:** Factors affecting odds of Bayley-III Language Scores < 85—by primary language (multivariate analysis).

Variable	Adjusted Odds Ratio (95% CI ^1^)
English or French as a primary language	0.84 (0.68, 1.04)
Number of languages exposed to (vs. one)	
Two	1.45 (1.21, 1.73)
More than two	1.62 (1.18, 2.23)
Gestational age	0.93 (0.88, 0.98)
Caregiver 1 education (vs. completed post-secondary)	
Did not complete high school	2.16 (1.67, 2.80)
Completed high school only	1.58 (1.34, 1.87)
Single caregiver	1.69 (1.23, 2.32)
Rural residence	1.04 (0.83, 1.31)
Hearing aids or cochlear implants	2.49 (1.37, 4.49)
Bayley-III Motor Composite < 85	3.22 (2.64, 3.93)
Bayley-III Cognitive < 85	7.83 (6.04, 10.1)

^1^ 95% confidence interval.

**Table 6:** Factors affecting odds of Bayley-III Language Composite Scores < 85—by language of administration (multivariate analysis).

Variable	Adjusted Odds Ratio (95% CI ^1^)
Language of administration	
French vs. English	1.05 (0.88, 1.26)
Interpreter vs. English	1.13 (0.76, 1.67)
Number of languages exposed to (vs. one)	
Two	1.53 (1.29, 1.81)
More than two	1.70 (1.24, 2.32)
Gestational age	0.92 (0.88, 0.98)
Caregiver 1 education (vs. completed post-secondary)	
Did not complete high school	2.12, (1.64, 2.76)
Completed high school only	1.55 (1.31, 1.84)
Single caregiver	1.62 (1.18, 2.23)
Rural residence	1.04 (0.83, 1.31)
Hearing aids or cochlear implants	2.42 (1.33, 4.38)
Motor Composite < 85	3.25 (2.65, 3.97)
Cognitive < 85	7.91 (6.10, 10.3)

^1^ 95% confidence interval.

## Data Availability

Data is available upon request but is not publicly available according to research ethics board and data sharing agreements.

## Supplementary Materials

The following supporting information can be downloaded at: https://www.mdpi.com/article/10.3390/children9071048/s1, Table S1. CNFUN Site Investigators and Steering Committee. The CNFUN Steering Committee and site-investigators are noted in this Supplementary Material.

## References

[1] Vohr B. (2013). Speech and Language Outcomes of Very Preterm Infants. Semin. Fetal Neonatal Med..

[2] van Noort-van der Spek I.L., Franken M.-C.J.P., Weisglas-Kuperus N. (2012). Language Functions in Preterm-Born Children: A Systematic Review and Meta-Analysis. Pediatrics.

[3] Barre N., Morgan A., Doyle L.W., Anderson P.J. (2011). Language Abilities in Children Who Were Very Preterm and/or Very Low Birth Weight: A Meta-Analysis. J. Pediatr..

[4] Synnes A., Luu T.M., Moddemann D., Church P., Lee D., Vincer M., Ballantyne M., Majnemer A., Creighton D., Yang J. (2017). Determinants of Developmental Outcomes in a Very Preterm Canadian Cohort. Arch. Dis. Child.-Fetal Neonatal Ed..

[5] Eadie P., Bavin E.L., Bretherton L., Cook F., Gold L., Mensah F., Wake M., Reilly S. (2021). Predictors in Infancy for Language and Academic Outcomes at 11 Years. Pediatrics.

[6] Johnson C.J., Beitchman J.H., Brownlie E.B. (2010). Twenty-Year Follow-Up of Children with and Without Speech-Language Impairments: Family, Educational, Occupational, and Quality of Life Outcomes. Am. J. Speech-Lang. Pat..

[7] Conti-Ramsden G., Durkin K. (2012). Postschool Educational and Employment Experiences of Young People with Specific Language Impairment. Lang. Speech Hear Serv. Sch..

[8] Schoon I., Parsons S., Rush R., Law J. (2010). Children’s Language Ability and Psychosocial Development: A 29-Year Follow-up Study. Pediatrics.

[9] Gianní M.L., Picciolini O., Ravasi M., Gardon L., Vegni C., Fumagalli M., Mosca F. (2006). The Effects of an Early Developmental Mother–Child Intervention Program on Neurodevelopment Outcome in Very Low Birth Weight Infants: A Pilot Study. Early Hum. Dev..

[10] Meijssen D., Wolf M.-J., Koldewijn K., Houtzager B.A., van Wassenaer A., Tronick E., Kok J., van Baar A. (2010). The Effect of the Infant Behavioral Assessment and Intervention Program on Mother-Infant Interaction after Very Preterm Birth: Effect of an Early Intervention on Mother-Infant Interaction after Very Preterm Birth. J. Child Psychol. Psyc..

[11] Nordhov S.M., Rønning J.A., Ulvund S.E., Dahl L.B., Kaaresen P.I. (2011). Early Intervention Improves Behavioral Outcomes for Preterm Infants: Randomized Controlled Trial. Pediatrics.

[12] Ravn I.H., Smith L., Lindemann R., Smeby N.A., Kyno N.M., Bunch E.H., Sandvik L. (2011). Effect of Early Intervention on Social Interaction between Mothers and Preterm Infants at 12 Months of Age: A Randomized Controlled Trial. Infant. Behav. Dev..

[13] Vohr B., Wright L.L. (2011). Follow-up Care of High-Risk Infants. Pediatrics.

[14] Bayley N. (2006). Manual for the Bayley Scales of Infant and Toddler Development.

[15] Kinsella-Ritter A., Gibson F.L., Wyver S. (2009). The Clinical Use of the Bayley Scales of Infant and Toddler Development, Third Edition (Bayley-III) in Australia. Aust. Educ. Dev. Psychol..

[16] McHenry M.S., Oyungu E., Yang Z., Hines A.C., Ombitsa A.R., Vreeman R.C., Abubakar A., Monahan P.O. (2021). Cultural Adaptation of the Bayley Scales of Infant and Toddler Development, 3rd Edition for Use in Kenyan Children Aged 18–36 Months: A Psychometric Study. Res. Dev. Disabil.

[17] Lowe J.R., Nolen T.L., Vohr B., Adams-Chapman I., Duncan A.F., Watterberg K. (2013). Effect of Primary Language on Developmental Testing in Children Born Extremely Preterm. Acta Paediatr..

[18] Adams-Chapman I., Bann C., Carter S.L., Stoll B.J. (2015). Network, for the N.N.R. Language Outcomes among ELBW Infants in Early Childhood. Early Hum. Dev..

[19] Von Elm E., Altman D.G., Egger M., Pocock S.J., Gøtzsche P.C., Vandenbroucke J.P., Initiative S. (2008). The Strengthening the Reporting of Observational Studies in Epidemiology (STROBE) Statement: Guidelines for Reporting Observational Studies. J. Clin. Epidemiol..

[20] Canadian Neonatal Follow-Up Network 18-Month Corrected Age Assessment Manual 2018 Version 6. April 2018..

[21] Rosenbaum P., Paneth N., Leviton A., Goldstein M., Bax M., Damiano D., Dan B., Jacobsson B. (2007). A Report: The Definition and Classification of Cerebral Palsy April 2006. Dev. Med. Child Neurol. Suppl..

[22] Sansavini A., Zuccarini M., Gibertoni D., Bello A., Caselli M.C., Corvaglia L., Guarini A. (2021). Language Profiles and Their Relation to Cognitive and Motor Skills at 30 Months of Age: An Online Investigation of Low-Risk Preterm and Full-Term Children. J. Speech Lang. Hear Res..

[23] Loeb D.F., Imgrund C.M., Lee J., Barlow S.M. (2020). Language, Motor, and Cognitive Outcomes of Toddlers Who Were Born Preterm. Am. J. Speech-Lang. Pat..

[24] Wang M.V., Lekhal R., Aaro L.E., Holte A., Schjolberg S. (2014). The Developmental Relationship between Language and Motor Performance from 3 to 5 Years of Age: A Prospective Longitudinal Population Study. BMC Psychol..

[25] Conti-Ramsden G., Durkin K. (2016). What Factors Influence Language Impairment Considering Resilience as Well as Risk. Folia Phoniatr. Logo.

[26] American Speech-Language Hearing Association (n.d.) Bilingual Service Delivery (Practice Portal). www.asha.org/Practice-Portal/Professional-Issues/Bilingual-Service-Delivery/.

[27] Madaschi V., Mecca T.P., Macedo E.C., Paula C.S. (2015). Bayley-III Scales of Infant and Toddler Development: Transcultural Adaptation and Psychometric Properties*. Paid. Ribeirão Preto.

[28] Duncan A.F., Watterberg K.L., Nolen T.L., Vohr B.R., Adams-Chapman I., Das A., Lowe J. (2012). Effect of Ethnicity and Race on Cognitive and Language Testing at Age 18-22 Months in Extremely Preterm Infants. J. Pediatr..

[29] Reilly S., Wake M., Ukoumunne O.C., Bavin E., Prior M., Cini E., Conway L., Eadie P., Bretherton L. (2010). Predicting Language Outcomes at 4 Years of Age: Findings From Early Language in Victoria Study. Pediatrics.

[30] Van Veen S., Remmers S., Aarnoudse-Moens C.S.H., Oosterlaan J., van Kaam A.H., van Wassenaer-Leemhuis A.G. (2019). Multilingualism Was Associated with Lower Cognitive Outcomes in Children Who Were Born Very and Extremely Preterm. Acta Paediatr..

[31] Werker J. (2012). Perceptual Foundations of Bilingual Acquisition in Infancy. Ann. N. Y. Acad. Sci..

[32] Portocarrero J.S., Burright R.G., Donovick P.J. (2007). Vocabulary and Verbal Fluency of Bilingual and Monolingual College Students. Arch. Clin. Neuropsychol. Off. J. Natl. Acad. Neuropsychol..

[33] Luo L., Luk G., Bialystok E. (2010). Effect of Language Proficiency and Executive Control on Verbal Fluency Performance in Bilinguals. Cognition.

[34] Li H., Wu D., Yang J., Xie S., Chang C., Luo J. (2022). Bilinguals Have More Effective Executive Function: Evidence from an FNIRS Study of the Neural Correlates of Cognitive Shifting. Int. J. Bilingual..

[35] Akhtar N., Menjivar J.A. (2012). Chapter 2 Cognitive and Linguistic Correlates of Early Exposure to More than One Language. Adv. Child Dev. Behav..

[36] Nosarti C., Rushe T.M., Woodruff P.W.R., Stewart A.L., Rifkin L., Murray R.M. (2004). Corpus Callosum Size and Very Preterm Birth: Relationship to Neuropsychological Outcome. Brain J. Neurol..

[37] Murray A.L., Scratch S.E., Thompson D.K., Inder T.E., Doyle L.W., Anderson J.F.I., Anderson P.J. (2014). Neonatal Brain Pathology Predicts Adverse Attention and Processing Speed Outcomes in Very Preterm and/or Very Low Birth Weight Children. Neuropsychology.

[38] Woods P.L., Rieger I., Wocadlo C., Gordon A. (2014). Predicting the Outcome of Specific Language Impairment at Five Years of Age through Early Developmental Assessment in Preterm Infants. Early Hum. Dev..

[39] Paul L.K. (2010). Developmental Malformation of the Corpus Callosum: A Review of Typical Callosal Development and Examples of Developmental Disorders with Callosal Involvement. J. Neurodev. Disord..

[40] Vandormael C., Schoenhals L., Hüppi P.S., Filippa M., Tolsa C.B. (2019). Language in Preterm Born Children: Atypical Development and Effects of Early Interventions on Neuroplasticity. Neural. Plast..

[41] Shinya Y., Kawai M., Niwa F., Kanakogi Y., Imafuku M., Myowa M. (2022). Cognitive Flexibility in 12-Month-Old Preterm and Term Infants Is Associated with Neurobehavioural Development in 18-Month-Olds. Sci. Rep..

[42] Jansson-Verkasalo E., Ruusuvirta T., Huotilainen M., Alku P., Kushnerenko E., Suominen K., Rytky S., Luotonen M., Kaukola T., Tolonen U. (2010). Atypical Perceptual Narrowing in Prematurely Born Infants Is Associated with Compromised Language Acquisition at 2 Years of Age. BMC Neurosci..

[43] Speech-Language and Audiology Canada Learning an Additional Language in the Context of Language Disorder 2021. https://www.sac-oac.ca/sites/default/files/Position_Statement_Learning_an_Additional_Language_in_the_Context_of_Language_Disorder_EN.pdf.

[44] Grunau R.V.E., Kearney S.M., Whitfield M.F. (1990). Language Development at 3 Years in Pre-term Children of Birth Weight below 1000 g. Int. J. Lang. Comm. Dis..

[45] Sanchez K., Spittle A.J., Cheong J.L., Thompson D.K., Doyle L.W., Anderson P.J., Morgan A.T. (2019). Language in 2-Year-Old Children Born Preterm and Term: A Cohort Study. Arch. Dis. Child.

