# Supplementary material for: Impact of Differing Language Background Exposures on Bayley-III Language Assessment in a National Cohort of Children Born Less than 29 Weeks’ Gestation
Source: Children (Basel). 2022 Jul 14;9(7):1048. doi: 10.3390/children9071048 (PMC9316512; doi:10.3390/children9071048)
Supplement: Supplementary file 1 [file children-09-01048-s001.zip › children-1780806-supplementary.pdf]

Table S1. CNFUN Site Investigators and Steering Committee

**CNFUN**
Steering Committee: Anne Synnes MDCM MHSc (Past Director), Thuy Mai Luu MD MSc (Director), Jehier Afifi MB BCh MSc (Co-Director), Rudaina Banihani MD, Jill Zwicker PhD OT, Lindsay Colby RN BScN MSN, Matthew Hicks MD PhD, M. Florencia Ricci MD PhD, Karen Thomas MD, Marie-Noelle Simard PhD
Site Investigators: Thevanisha Pillay MD, Victoria General Hospital, Victoria, British Columbia; Shelagh Anson MD, British Columbia Children's Hospital, Vancouver, British Columbia; Rebecca Sherlock MD, Surrey Memorial Hospital, Surrey, British Columbia; Miroslav Stavel MD, Anitha Moodley MD, Royal Columbian Hospital, New Westminister, British Columbia; Leonora Hendson MD, Alberta Children's Hospital/Foothills Medical Centre, Calgary, Alberta; Amber Reichert MD, Glenrose Rehabilitation Hospital, Edmonton, Alberta; Diane Moddemann MD MEd, Cecilia de Cabo MD, Winnipeg Health Sciences Centre, St. Boniface General Hospital, Winnipeg, Manitoba; Judy Seesahai MD, Windsor Regional Hospital, Windsor, Ontario; Sarah McKnight MD, Kingston General Hospital, Kingston, Ontario; Kevin Coughlin MD, Children's Hospital London Health Sciences Centre, London, Ontario; Linh Ly, MD, Hospital for Sick Children, Toronto, Ontario; Kamini Raghuram MD, Mount Sinai Hospital, Toronto, Ontario; Karen Thomas MD, Hamilton Health Sciences Centre, Hamilton, Ontario; Paige Church MD, Rudaina Banihani MD, Sunnybrook Health Sciences Centre, Toronto, Ontario; Kim-Anh Nguyen MD, Jewish General Hospital, Montréal, Québec; May Khairy, MD, Marc Beltempo MD, Montréal Children's Hospital, Montréal, Québec; Thuy Mai Luu MD MSc, Centre Hospitalier Universitaire Sainte-Justine, Montréal, Québec; Alyssa Morin MD, Centre Hospitalier Universitaire de Sherbrooke, Sherbrooke, Québec; Sylvie Bélanger MD, Centre Hospitalier Universitaire de Québec, Québec City, Québec; Jehier Afifi MB BCh MSc, IWK Health Centre, Halifax, Nova Scotia.
